# Predictive and prognostic value of Matrix metalloproteinase (MMP) - 9 in neoadjuvant chemotherapy for triple-negative breast cancer patients

**DOI:** 10.1186/s12885-018-4822-7

**Published:** 2018-09-21

**Authors:** Ruo-Xi Wang, Sheng Chen, Liang Huang, Zhi-Ming Shao

**Affiliations:** 1 Department of Breast Surgery, Fudan University Shanghai Cancer Center/Cancer Institute, 399 Ling-Ling Road, Shanghai, 200032 People’s Republic of China; 2 Department of Oncology, Shanghai Medical College, Fudan University, Shanghai, People’s Republic of China; 3 Institutes of Biomedical Science, Fudan University, Shanghai, People’s Republic of China

**Keywords:** Breast cancer, Neoadjuvant chemotherapy, MMP-9, Pathological response, Prognostic marker

## Abstract

**Background:**

This study aimed to investigate the clinical utility of serum and histological MMP-9 detection during neoadjuvant chemotherapy (NAC) for triple-negative breast cancer (TNBC).

**Methods:**

A total of 303 TNBC patients who underwent weekly paclitaxel plus carboplatin treatments followed by surgical resection were included in this study. Enzyme-linked immunosorbent assay (ELISA) was used to detect the serum level of Matrix metalloproteinase-9 (sMMP-9) at baseline and prior to surgery. Immunohistochemistry was used to detect histological MMP-9 (hMMP-9) expression in patients with residual tumors after NAC. The value of MMP-9 to predict the response to NAC and patient survival was studied.

**Results:**

Of the 303 patients, 103 (34.0%) patients experienced pathological complete response (pCR) after completion of NAC. Univariate and multivariate analyses revealed that the relative change in sMMP-9, rather than sMMP-9 at baseline or surgery, had a remarkable predictive value for pCR. Each 1 ng/ml decrease in sMMP-9 after NAC was shown to result in a 0.3% increase in pCR rate. Additionally, in survival analyses, hMMP-9 expression in residual tumors was independently correlated with disease-free survival for non-pCR responders (*P* < 0.001).

**Conclusions:**

Our findings indicate that monitoring serum MMP-9 and detection of histological MMP-9 could help identify TNBC patients who will respond to NAC and will display varying risks of disease relapse. MMP-9 may serve as a predictive and prognostic biomarker for tailoring and modifying the NAC strategy for TNBC.

## Background

Triple-negative breast cancer (TNBC) is a type of breast cancer that lacks the expression of estrogen receptor (ER), progesterone receptor (PgR) and human epidermal growth factor receptor-2 (HER2). It comprises 15–20% of all breast cancers and has an aggressive tumor biology [1]. For locally advanced TNBC, neoadjuvant chemotherapy (NAC) followed by definitive surgery is a standard of care. It is also an option for early-stage TNBC to increase the chance of breast-conserving surgery. The use of NAC has also provided insight into tumor biology and differential responses to treatment. The result of NAC, often evaluated by pathological response according to surgical specimens, has significantly impacted patient survival. The patients who have achieved pathological complete response (pCR) have a relatively lower risk of disease relapse or death compared to those who have residual disease after NAC [2, 3].

In a neoadjuvant setting, although TNBCs have a higher pCR rate compared with other subtypes [4], it is not clearly translated into an improved overall survival [5]. This is possibly due to a poor outcome of non-pCR responders. Since TNBC has proved to be a heterogeneous disease comprising subtypes with different biological behaviors and clinical outcomes [6, 7], strategies that identify candidates who respond differently or display a better prognosis as a result of NAC are needed. Several pathological biomarkers have been investigated including P53, cytokeratin (CK) 5/6, CK14, epidermal growth factor receptor (EGFR), and Ki-67 [6, 7]. However, clinically applicable biologic markers of predictive or prognostic value are still limited.

Matrix metalloproteinases (MMPs) are a family of zinc-dependent endopeptidases that are involved in the degradation of the extracellular matrix (ECM) [8]. Several MMPs share a large amount of common structural and functional similarities and have been found to play key roles in cancer invasion and metastasis, angiogenesis and tumorigenesis [9–11]. Recently, a meta-analysis comprising 28 studies indicated that MMP-9 expression in serum and tumor tissue acts as a predictor for worse prognosis in breast cancer [12]. However, few attempts have been made to investigate the predictive and prognostic value of MMP-9 on TNBCs specifically. This study was designed to demonstrate the practical utility of MMP-9 detection in a neoadjuvant setting and to establish a new strategy to identify subgroups of TNBC patients with different risk.

## Methods

### Study population

In this study, we selected 303 patients with TNBC according to inclusive and exclusive criteria reported previously [13]. The cut-off values of 1% of positive tumor cells were used for ER positivity and PgR positivity. Circumferential membrane-bound staining of 0, 1+,2+ or 3+ were used to evaluated HER2 expression. Positivity for HER2 (HER2+) was considered as 3 + using IHC or as positive on fluorescence in situ hybridization (FISH). Only tumors with negative expression of ER, PgR and HER2 were considered as TNBC. All patients were confirmed with invasive breast cancer by core needle biopsy. The regimen of NAC was weekly PC (paclitaxel plus carboplatin) followed by surgical resection at Fudan University Shanghai Cancer Center between January 2009 and July 2015. Our study was approved by the independent ethical committee/institutional review board of our center (Shanghai Cancer Center Ethical Committee). All patients gave their written informed consent before inclusion in this study.

The NAC regimen was paclitaxel (80 mg/m^2^) and carboplatin (AUC 2 mg*min/ml) on days 1, 8, and 15 of a 28-day cycle for 6 cycles. Following NAC, all patients received mastectomy or breast conserving surgery to remove the primary tumor, and axillary lymph nodes dissection to remove lymph nodes. After pathological evaluation of surgical specimens, patients with complete response (pCR) would receive two additional cycles of PC, whereas non-pCR responders would receive three cycles of anthracycline-based chemotherapy. The performing of radiation therapy was at the discretion of the treating radiologist.

### Response evaluation

Response Evaluation Criteria in Solid Tumors (RECIST) version 1.1 [14] was used in evaluation of clinical response based on MRI and ultrasound examinations. Miller-Payne (MP) grading system [15] was used to evaluate pathological response. Pathological complete response was defined as no residual invasive cancer in either the breast or lymph nodes.

### ELISA and immunohistochemistry

**Fig. 1:**
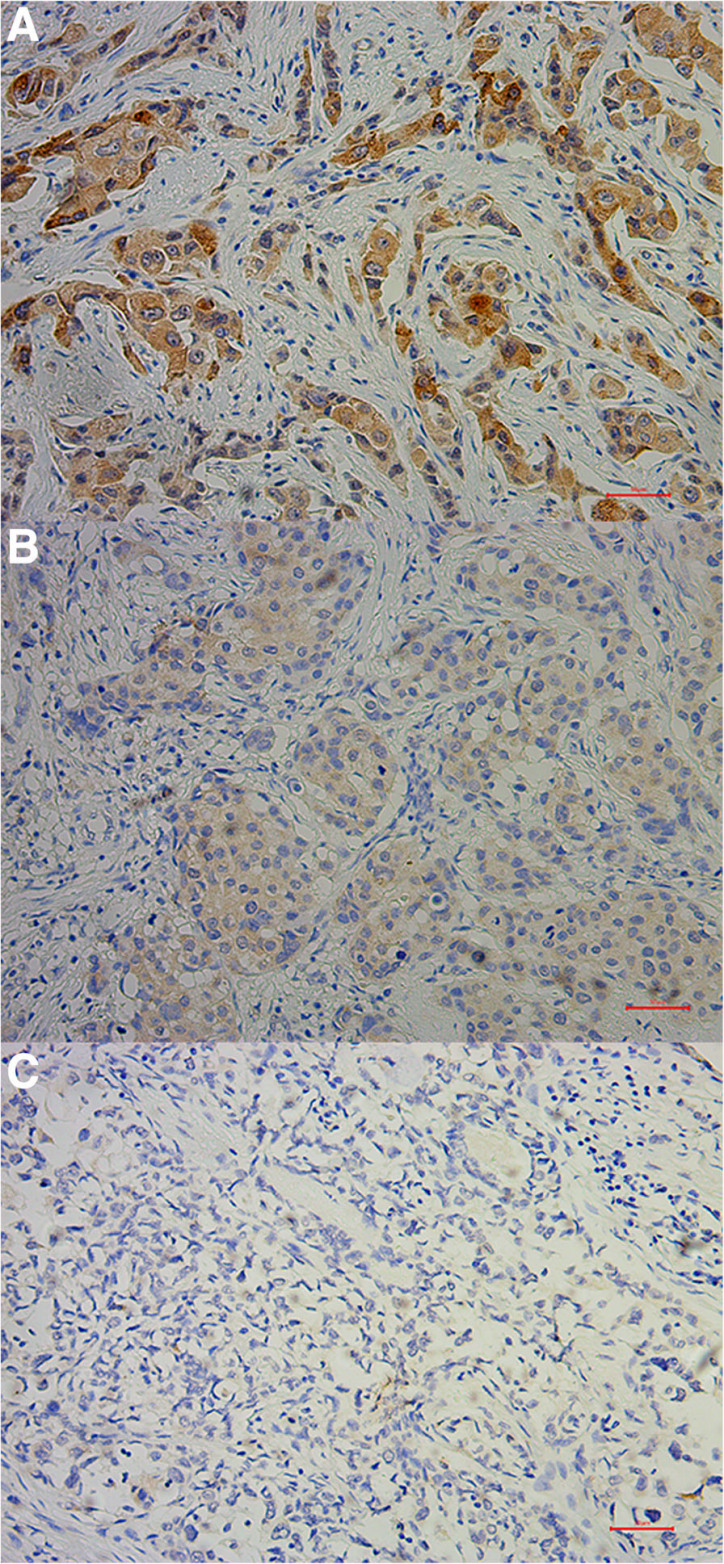
Immunohistochemical staining of MMP-9 in post-NAC samples of breast cancer. MMP-9 protein was detected with immunohistochemistry (IHC) using paraffin-embedded breast cancer samples collected after neoadjuvant chemotherapy. **a** Representative IHC images of strongly positive MMP-9 staining (200X). **b** Representative IHC images of moderate MMP-9 staining. **c** Representative IHC images of negative MMP-9 staining (200X). Scale bar: 50 μm

Serum samples were obtained prior to the start of NAC and prior to surgery. Patients’ blood was drawn using standard protocols and was collected in the absence of anticoagulant. Sera were then separated through centrifugation. All sera sample were stored at − 80 °C. No more than two freeze–thaw cycles were allowed before the procedure of enzyme-linked immunosorbent assay (ELISA). The MMP-9 level in each sample was then measured with antibody from Proteintech (10375–2-AP, Rosemont, IL, USA). Measurement of MMP-9 was marked as the average level of the repeated results (three times). The mean standard error was 0.511. Expression of MMP-9 protein in the tissue sections from surgical specimens after NAC were evaluated through Immunohistochemistry (IHC) with antibody from Cell Signaling Technology (#13667, Danvers, MA, USA). Sections of spleen tissue were used as a positive control. Two pathologists reviewed and measured the results of IHC independently using the H score. The intensity of positivity was scored as no staining (0), weak staining (+ 1), moderate staining (+ 2) and intense staining (+ 3). In this study, we defined patients with an H score of 0–100 as MMP-9 negative (−), 100–200 as moderately positive (+) and 200–300 as strongly positive (++) (Fig. 1).

### Statistical analysis

**Fig. 2:**
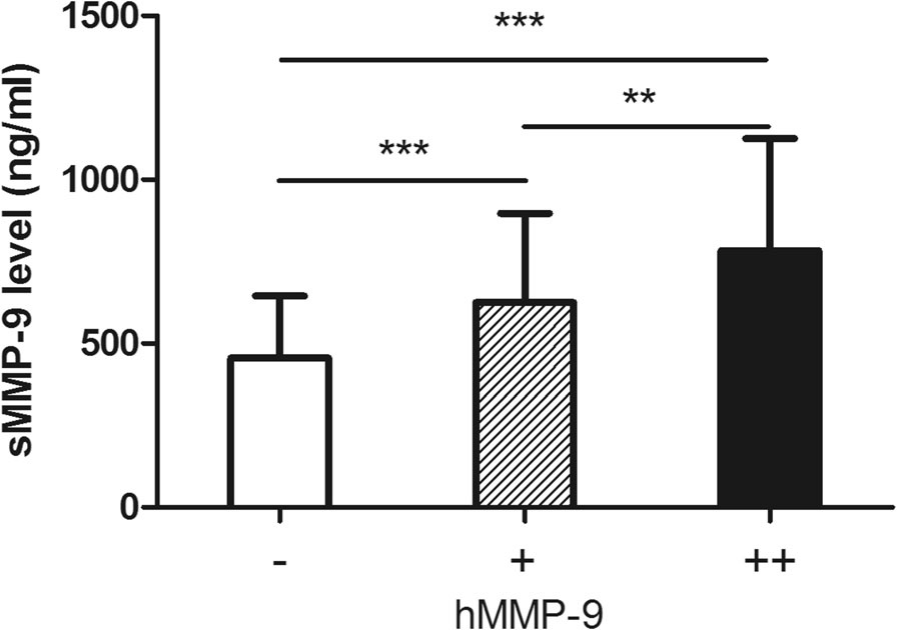
Correlation between serum and histological MMP-9 in non-pCR patients. A higher serum MMP-9 (sMMP-9) level is more frequently observed in patients with higher positivity of histological MMP-9 level (hMMP-9), ***P < 0.001, **P < 0.01

**Fig. 3:**
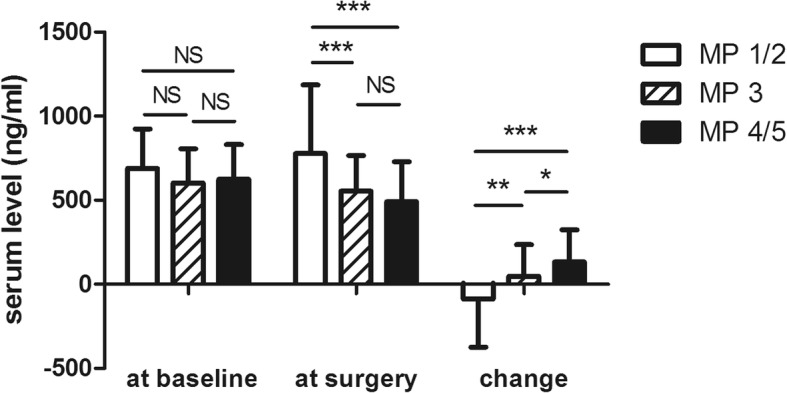
Correlation between serum MMP-9 value and Miller-Payne (MP) grades. MMP-9 change after NAC was significantly correlated to response to NAC. The mean absolute changes (ng/ml) in serum MMP-9 were − 88.30 ± 286.40, 47.70 ± 188.60 and 133.00 ± 190.40 in patients with a poor response (MP 2/1), a partial response (MP 3) and an ideal response (MP 5/4), respectively. ***P < 0.001, **P < 0.01, *P < 0.05

Two-tailed Student’s T test was used to compare means between two groups (in Fig. 2 and Fig. 3). Relationship between patient characteristics and response were evaluated by Chi-squared test. Logistic regression model with the forward selection of variables was used to identify the independent predictors of treatment response. DFS was calculated from the date of surgery to disease relapse (local or distant relapse or death from any cause). Patients without events or death were censored at the last follow-up. Cox regression model was used to investigate the prognostic variables. Kaplan–Meier survival curves, together with the log-rank test was performed to show the difference between risk groups. All statistical tests were two-sided, and *P* values less than 0.05 were considered significant. SPSS (version 19.0, SPSS Company, Chicago, IL, USA) software was used to perform the Statistical analyses.

## Results

### Patient characteristics

Patient characteristics are shown in Table 1. Among 303 patients in this study, the median age was 50 years (range, 27–74 years). A total of 52.5% of patients were pre-menopausal at diagnosis. All patients were diagnosed with stage II or III disease, and 82.8% of them had positive lymph nodes prior to NAC. Ki-67 expression of biopsy specimens was also evaluated, and 64.7% of patients had a Ki-67 level greater than 20%.

**Table 1:** Patient characteristics and observed pathological complete response (pCR)

Characteristics	Number of patients	Number of pCR (%)	Chi-squared test *P* value	Multivariate *P* value
Age			0.564	NS
< 40	60	23 (38.3)		
40–59	194	66 (34.0)		
60+	49	14 (28.6)		
Menopausal status			0.817	NS
Pre	159	55 (34.6)		
Post	144	48 (33.3)		
Tumor size at baseline			0.026	0.030
T2	150	62 (41.3)		
T3	100	28 (28.0)		
T4	53	13 (24.5)		
Node status at baseline			0.917	NS
-	52	18 (34.6)		
+	251	85 (33.9)		
Histology at baseline			0.263	NS
Invasive ductal carcinoma	224	82 (36.6)		
Invasive (mixed) carcinoma	62	17 (27.4)		
Others	17	4 (23.5)		
Ki-67 expression at baseline			< 0.001	0.001
< 20%	107	22 (20.6)		
≥ 20%	196	81 (41.3)		
sMMP-9 at baseline (ng/ml)			0.519	NS*
Low (< 505.5)	101	34 (33.7)		
Intermediate (505.5–712.8)	100	38 (38.0)		
High (> 712.8)	102	31 (30.4)		
sMMP-9 at surgery (ng/ml)			0.020	0.043*
Low (< 423.2)	100	42 (42.0)		
Intermediate (423.2–612.3)	102	37 (36.3)		
High (> 612.3)	101	24 (23.8)		
sMMP-9 decrease (ng/ml)			< 0.001	0.003*
Low (< 28.3)	100	24 (24.0)		
Intermediate (28.3–143.8)	101	29 (28.7)		
High (> 143.8)	102	50 (49.0)		
Clinical response			0.172	NS
CR/PR	102	40 (39.2)		
SD/PD	201	63 (31.3)		

*sMMP-9 was studied in the multivariate analysis as linearly variable

### MMP-9 expression

Serum MMP-9 (sMMP-9) was measured by ELISA at two time points: prior to the start of NAC (at baseline), and prior to surgery (at surgery). The median levels of sMMP-9 at baseline and surgery were 607.2 ng/ml (range: 241.2 ng/ml - 1172.4 ng/ml) and 513.5 ng/ml (range: 120.4 ng/ml - 1886.7 ng/ml), respectively. The change in sMMP-9 level was calculated, and the median reduction was 82.2 ng/ml (range: − 878.3 - 629.5).

Histological MMP-9 (hMMP-9) was measured by IHC on surgical specimens of residual tumors. hMMP-9 data were available for 200 patients with residual tumors after NAC (non-pCR responders). According to the H-score, 81 patients (40.5%) were considered as hMMP-9 negative (−), whereas 55 patients (27.5%) were considered as weakly or moderately positive (+). Sixty-four patient samples (32.0%) were considered to be strongly positive (++) for hMMP-9. We also studied the correlation between sMMP-9 and hMMP-9. Higher sMMP-9 level (at surgery) is more frequently observed in patients with a higher positivity of hMMP-9, indicating a concordance between serum and histological expression (Fig. 2).

### sMMP-9 and treatment response

Of the 303 patients, 103 (34.0%) experienced pCR after completion of NAC. Table 1 shows the results of the Chi-squared test and multivariate logistic regression analysis for pCR predictors. Correlations between pCR and clinical or pathological variables, including patient age, menopausal status, primary tumor size, node status, stage, Ki-67 value, sMMP-9 and clinical response, were determined. The sMMP-9 category was defined according to the tertile cutoff points. A lower level of sMMP-9 at surgery and a higher level of sMMP-9 decrease were correlated with a higher possibility of achieving pCR; however, sMMP-9 at baseline was not a predictor of pCR (*P* = 0.519). In multivariate analysis, the decrease in sMMP-9 independently correlated with pCR as a continuous variable (*P* = 0.003, HR = 1.003, 95% CI: 1.002–1.005). Each 1 ng/ml decrease in the sMMP-9 level after NAC resulted in a 0.3% increase in the pCR rate. sMMP-9 at surgery also tended to be correlated with pCR (*P* = 0.046, HR = 0.997, 95%CI: 0.994–0.998). Tumor size and Ki-67 expression at baseline were also independent predictors of pCR (*P* = 0.030, HR = 0.536 for T3, and HR = 0.430 for T4, T2 as reference; and *P* = 0.001, HR = 2.826 for high Ki67, low Ki-67 as reference, respectively).

The correlation between the sMMP-9 level and tumor regression (according to MP grades) is shown in Fig. 3. A significant decrease in sMMP-9 after NAC was most frequently observed in patients with a relatively better response. The mean absolute decrease in the sMMP-9 value was 133.0 ng/ml in patients with an ideal response (MP 5/4), 47.7 ng/ml in patients with a partial response (MP 3), and − 88.3 ng/ml in patients with a poor response (MP 2/1).

### MMP-9 and patient survival

The median follow-up time for all patients was 45 months. Among the 103 pCR patients, only 4 developed disease recurrence or metastasis. However, non-pCR responders had a relatively poor survival, with 65 (32.5%) cases of event or death.

**Fig. 4:**
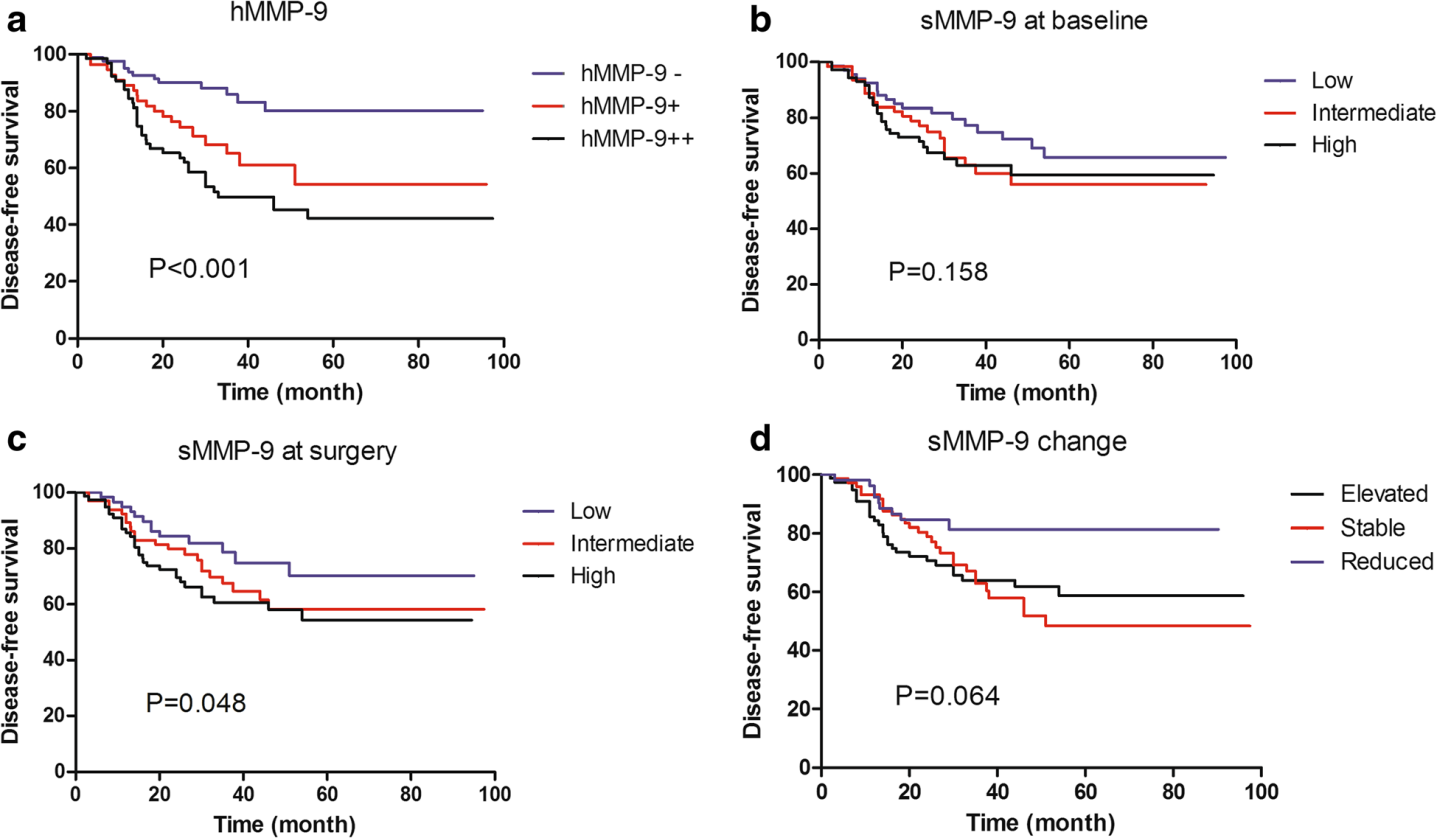
Cumulative disease-free survival of non-pCR patients according to MMP-9 levels after NAC. High expression of hMMP-9 was significantly correlated with an unfavorable outcome (*P* < 0.001). **a** Disease-free survival according to histological expression of MMP-9 protein (hMMP-9); **b** Disease-free survival according to serum MMP-9 (sMMP-9) at baseline; **c** Disease-free survival according to sMMP-9 at surgery; **d** Disease-free survival according to sMMP-9 change

Among the 200 non-pCR responders, univariate survival analysis was performed to assess the prognostic value of each variable. Residual tumor size (*P* < 0.001), residual node involvement (*P* < 0.001), vascular invasion (*P* = 0.035), residual tumor Ki-67 (*P* = 0.001), sMMP-9 at surgery (*P* = 0.008), sMMP-9 change (*P* = 0.019) and hMMP-9 (*P* < 0.001) were significant predictors of DFS and were entered into the multivariate Cox regression model with forward selection. Patient age, menopausal status, primary tumor size, primary node status, primary Ki-67 expression, residual tumor grade and sMMP-9 at baseline were not significant variables. In the Cox model (Table 2), hMMP-9 expression was an independent prognostic value of DFS (hMPP-9+, HR = 2.637, 95% CI: 1.341–5.183; hMMP-9++, HR = 2.832, 95% CI: 1.348–5.950, hMMP-9- was used as a reference; *P* = 0.004). Residual node involvement (*P* < 0.001) and Ki-67 value (*P* = 0.018) were also independent predictors of patient outcome. Better survival was more frequently observed in patients with a lower expression of hMMP-9, a lower Ki-67 value and fewer involved nodes. The distributions of survival curves by categorical MMP-9 are shown in Fig. 4. Compared to sMMP-9, hMMP-9 expression can better identify patients with different risk of relapse or death (Log-rank test *P* < 0.001). The observed 3-year RFS for hMMP-9 -, +, and ++ was 88.2% (± 3.8%), 65.1% (± 7.2%), and 49.8% (± 6.4%), respectively.

**Table 2:** Univariate and multivariate survival analysis of non-pCR patients

Factors	Disease-free survival		
	Univariate	Multivariate	
	P	P	HR (95% CI)
Age			
< 40 vs. 40–60 vs. ≥ 60	0.062	–	–
Menopausal status			
Pre vs. Post	0.150	–	–
Initial tumor status			
T2 vs. T3 vs. T4	0.104	–	–
Residual tumor size			
≤ 2 cm vs. 2-5 cm vs. > 5 cm	< 0.001	NS	–
Residual involved nodes			
0 vs. 1–3 vs. ≥4	< 0.001	< 0.001	1.000
			1.409 (0.604–3.289)
			4.124 (1.948–8.732)
Vascular invasion			
Negative vs. Positive	0.035	NS	–
Grade			
I - II vs. III	0.322	–	–
Ki-67			
< 20% vs. ≥ 20%	0.001	0.018	1.909 (1.117–3.262)
sMMP-9 at baseline			
Linear	0.226	–	
sMMP-9 at surgery			
Linear	0.008	NS	–
sMMP-9 change			
Linear	0.019	NS	–
hMMP-9 in residual tumor			
- vs. + vs. ++	< 0.001	0.004	1.000
			2.637 (1.341–5.183)
			2.832 (1.348–5.950)

## Discussion

pCR following NAC implies the absence of residual invasive disease and strongly correlates with prolonged patient survival [2, 3]. Compared with other breast cancer subtypes, TNBC has a relatively higher possibility of achieving pCR; however, this advantage is not clearly translated into an improved overall survival due to the poor outcomes of non-pCR responders [5]. Therefore, the early identification of sensitive responders would have definitive value to base therapeutic decisions for TNBC patients. Since current prediction methods using measurements of clinical, pathological and radiological responses lack the necessary precision, the potential utility of serum biomarkers has begun to be investigated [16, 17]; however, there is little consensus in the field regarding the predictive and prognostic value of these biomarkers.

MMPs are a large family of proteolytic enzymes of the extracellular matrix and play important roles in extracellular matrix degradation, tumor cell invasion, metastasis and angiogenesis [8, 18, 19]. Among all MMPs, MMP-9 has been the most commonly studied. A recent study stated that GM-CSF and MMP-9 promote the protumorigenic effect of WAT progenitors on local and metastatic breast cancer [20]. Some other study noted that MMP-9 could be regulated by DNA methylation in breast cancer, which might resulted in the first step of metastasis through extracellular matrix degradation [21]. Previously, two meta-analyses published by Song et al., [22] and Ren et al., [12] have verified that MMP-9 overexpression predicted a higher risk for OS and RFS in patients with breast carcinoma; however, few studies have been reported referring the prognostic value of serum MMP-9. Tabouret E et al. reported that low MMP9 serum level was associated with better survival in HER2-positive patients treated with bevacizumab- and trastuzumab-based neoadjuvant chemotherapy [23]. In this study, we also provide evidence that MMP-9 is correlated with survival of patients with breast cancer; however, this is the first study that investigates the prognostic value of MMP-9 in a neoadjuvant setting. Since it is well-known that pCR responders would achieve a favorable outcome, we mainly focused on the value of MMP-9 in distinguishing non-pCR patients at different levels of recurrence or death risk. Various similar prognostic markers have been described in the literature. Meaningful factors include node status, ER, PgR, and Ki-67 [24–27]. Our study provides another useful biomarker that might help clinicians to discriminate patients with residual tumors into different risk groups for further individualized treatment.

Furthermore, response prediction is extremely important for NAC candidates, especially those with TNBC. If an accurate prediction of response/non-response can be made early in a patient’s treatment, the regimen could be modified accordingly; this is known as the response–guided treatment strategy, which may avoid unnecessary treatment-related toxicities and provide a better survival, regardless of pCR [28]. For predicted well-responders, additional NAC cycles with the same regimen are recommended; however, for predicted non-responders, an alternative regimen may be necessary [28]. In the present study, we have demonstrated that serum MMP-9 might correlated to treatment response. The change in MMP-9 level in serum was independently correlated to the possibility of achieving pCR, with each 1 ng/ml decrease in the sMMP-9 level after NAC resulting in a 0.3% increase in the pCR rate. Our findings also suggest that serum MMP-9 measurement may play a role in the response evaluation at any time point throughout the whole NAC period, considering its decreasing trend in patients with high-chemosensitivity (Fig. 2).

Our findings are mainly based on the serum detection of MMP-9, which may be a safer and more valid method to detect the expression of MMP-9. This approach represents a significant departure from existing models of response monitoring by using imaging-based metrics, also known as clinical response evaluation. Although several studies have indicated that MRI is an effective tool for predicting the response to NAC [29], the accuracy was lower when pCR was more rigorously defined [29] and varies with tumor subtype [30]. More importantly, the clinical response often lacks accuracy in the early prediction of pathologic response to neoadjuvant therapy [31]. Compared to other predictive markers such as Ki-67 [32], monitoring of serum markers such as MMP-9 is relatively convenient and easy to accept by patients. Thus, early detection of a MMP-9 change might be of some value as a complement in response evaluation method of NAC for TNBC. However, more prospective data are needed to further validate its predictive value. A combination of serum biomarkers, histological biomarkers and imaging-based metrics might be the mainstream in the future response evaluation of NAC.

Interestingly, the IHC detection of MMP-9 protein expression shows better prognostic value compared to serum MMP-9 in survival analysis for non-pCR responders, despite a good concordance between sMMP-9 and hMMP-9. It is believed that the residual chemotherapy-resistant disease after NAC is a surrogate for chemotherapy-resistant micrometastatic disease that can ultimately progress into clinically overt metastatic breast cancer. Since TNBCs are initially sensitive to NAC, the residual tumors are generally more aggressive, which results in unfavorable prognoses with short RFS and OS [33, 34]. Furthermore, several reports have suggested that the residual cancer cells in TNBC represent a heterogeneous group comprising subtypes with different outcomes [7]. Therefore, the expression of MMP-9 of residual cancer cells might reflect a subtype of TNBCs with more aggressive behavior, resulting in poor survival.

## Conclusion

We demonstrated a new strategy for response prediction and evaluation for TNBC patients undergoing chemotherapy. The monitoring of serum MMP-9 could help identify patients with favorable responses to NAC, which allows for the modification of NAC regimens. In non-pCR responders, the histological expression of MMP-9 of residual cancer cells is correlated with risk of relapse or death. In future prospective studies, MMP-9 might serve as a complement or alternative to traditional prediction and prognostic evaluating methodologies.

## Abbreviations

ELISA: Enzyme-linked immunosorbent assay
ER: Estrogen receptor
HER2: Human epidermal growth factor receptor-2
IHC: Immunohistochemistry
MMP: Matrix metalloproteinase
NAC: Neoadjuvant chemotherapy
pCR: Pathological complete response
PgR: Progesterone receptor
TNBC: Triple-negative breast cancer
